# Increase of CSF inflammatory profile in a case of highly active multiple sclerosis

**DOI:** 10.1186/s12883-019-1455-7

**Published:** 2019-09-26

**Authors:** R. Magliozzi, D. Marastoni, S. Rossi, M. Castellaro, V. Mazziotti, M. Pitteri, A. Gajofatto, S. Monaco, M. D. Benedetti, M. Calabrese

**Affiliations:** 1 Neurology B, Department of Neurosciences, Biomedicine and Movement Sciences, University of Verona, Policlinico G.B. Rossi, P.le L.A. Scuro, 10, 37134 Verona, Italy; 2 Division of Brain Sciences, Department of Medicine, Imperial College London, London, UK; 3 Department of Information Engineering, University of Padova, Padova, Italy

**Keywords:** Multiple sclerosis, CSF, Biomarkers, Cytokines, Chemokynes

## Abstract

**Background:**

Clinical and imaging follow-up coupled with cerebrospinal fluid (CSF) and possibly serum profiling could provide information on disease activity and disability evolution in multiple sclerosis patients.

**Case presentation:**

We describe the case of a relapsing-remitting MS patient whose history was characterized by failure of several therapeutic approaches and sustained disease activity. By using a highly sensitive immunoassay methodology, we examined protein expression of 70 inflammatory/cytotoxic molecules in two consecutive paired CSF and serum samples, obtained respectively in 2006 and 2013. At disease diagnosis, elevated CSF protein levels of an inflammatory pattern, including CXCL13, CXCL12, IFNγ, TNF, sTNFR1, IL8, sCD163, APRIL, BAFF, pentraxin III and MMP2 were found compared with a group of controls. At the second lumbar puncture, sustained disease activity was accompanied by considerable (more than 2 fold changes) increase expression of most of these inflammatory molecules while no significant changes in serum inflammatory markers were detected in the two consecutive serum samples.

**Conclusions:**

Elevated CSF protein expression of pro-inflammatory mediators, possibly specifically associated to GM demyelination, could remain stable or increase over time in patients with active multiple sclerosis. We underline the role of fluid analysis in understanding the pathophysiology of the disease and providing information on possible markers of disease activity and evolution.

**Electronic supplementary material:**

The online version of this article (10.1186/s12883-019-1455-7) contains supplementary material, which is available to authorized users.

## Background

Early identification of the specific immune-phenotype of each multiple sclerosis (MS) patient would be particularly important at a stage in which the most appropriate disease modifying agents could be early provided, in order to improve outcomes before the onset of irreversible tissue damage [1]. Up to now, only a detailed clinical and imaging follow-up analysis has helped to either obtain early disease diagnosis or to monitor the response to treatments. Advanced biomarker studies have highlighted the importance of serum/CSF profiling in predicting features and evolution of the disease and, possibly, the response to therapies. A highly inflammatory intrathecal profile is a characteristic of both relapsing-remitting [2–4] and progressive [2, 4] MS patients. We have recently shown that elevated and specific intrathecal pro-inflammatory pattern, including increased CSF levels of CXCL13, TNF, IFNγ, CXCL12, IL6, IL8 and IL10, BAFF, APRIL, LIGHT, TWEAK, sTNFR1, sCD163, MMP2 and pentraxin III, characterizes a subgroup of MS patients with higher levels of grey matter (GM) damage at the time of diagnosis [4].

We here describe a high and increasing over-time inflammatory CSF profile associated with sustained clinical and MRI disease activity in a MS patient.

## Case presentation

**Fig. 1:**
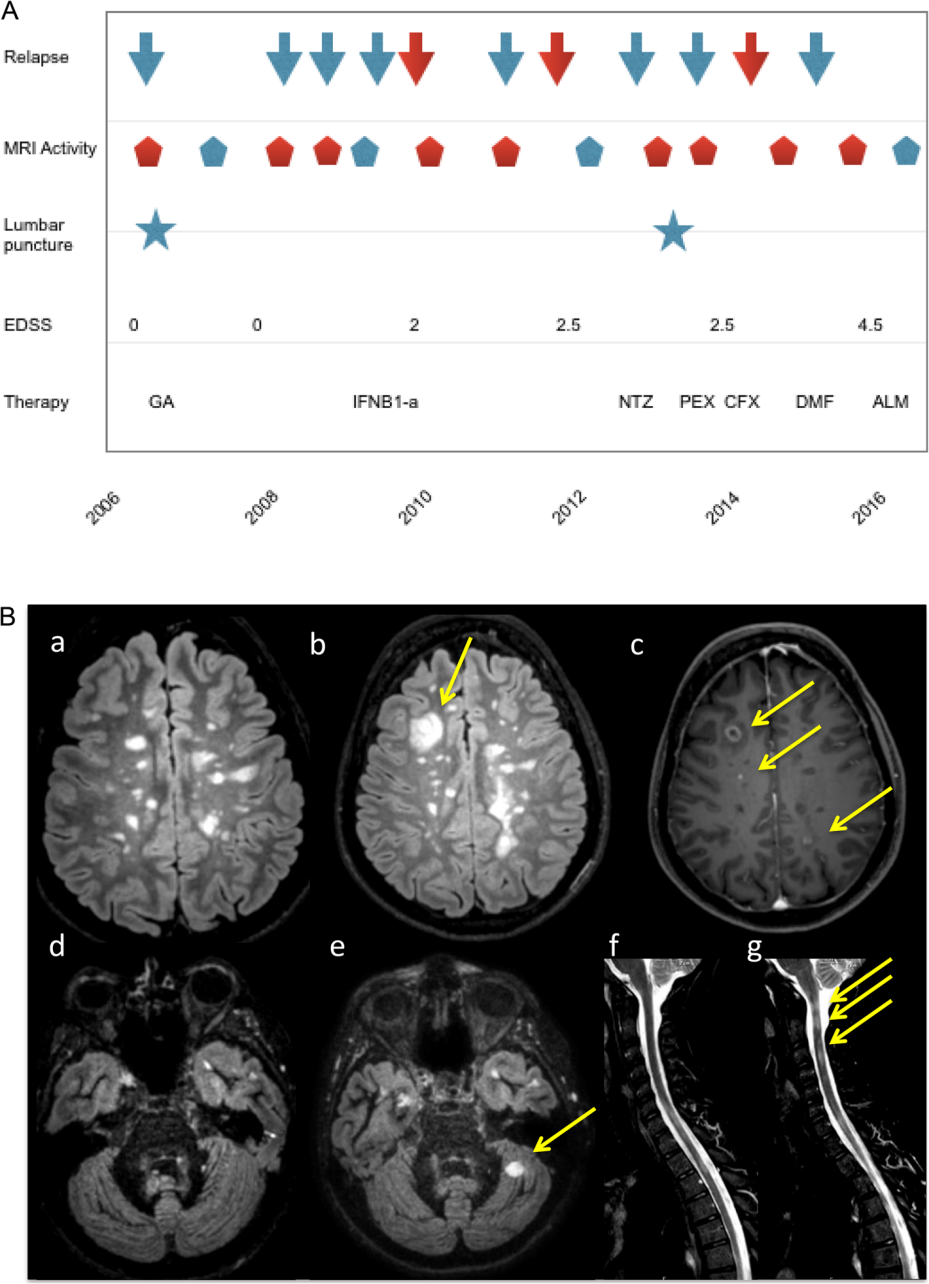
(A) Patient’s history was characterized by sustained disease activity. Red arrows correspond to relapses, which caused disability accumulation. Red pentagons correspond to disease activity at MRI follow up characterized by new T2 lesions or T1 Gd-enhancing lesions. Lumbar puncture was performed in 2006 and 2013. GA Glatiramer acetate; IFNB1a Interferon Beta-1a; NTZ Natalizumab; PEX Plasma Exchange; CFX cyclophosphamide; DMF Dimethyl-fumarate; ALM Alemtuzumab. (B) Brain 3 T-MRI obtained at the time of second lumbar puncture (a, d, f) and during a subsequent follow up of 2015 (b, c, e, g). Axial 3D Fluid Attenuated Inversion Recovery (FLAIR, a, b), post contrast T1w (c), 3D Double Inversion Recovery (d, e), spinal cord STIR (f, g) are shown. Arrows indicate new lesions

A 31-year-old woman was diagnosed with relapsing-remitting multiple sclerosis (RRMS) in December 2006 (first lumbar puncture, LP), according to revised McDonald criteria [5] after an onset with severe tactil hypoaesthesia in the left limbs. No oligoclonal bands (OCB) or increased intrathecal Ig synthesis were detected in the CSF; other possible causes were excluded. Her past medical history was unremarkable except for autoimmune thyroiditis in replacement therapy since 2004. At the time of diagnosis, brain MRI showed several T2-weighted hyperintense lesions in supratentorial and infratentorial white matter, some of them characterized by gadolinium (Gd)-enhancement on T1-weighted images The patient started glatiramer acetate treatment. In June and December 2008 she had two relapses (Fig. 1a); brain and spinal MRI confirmed sustained disease activity, characterized by new T2 weighted lesions and Gd + T1-weighted enhancing lesions. In 2009 her neurological examination showed only mild hyposthenia in right limb. The Expanded Disability Status Scale (EDSS) score was 2.0 [6]. Although switched to IFNB1-a therapy (22 mcg 3/week and then 44 mcg 3/week), she showed sustained disease activity with two annual relapses and new typical brain lesions.

From April to August 2013 (time of second LP) she was treated with Natalizumab, stopped due to a relapse with atypical brain MRI lesions. CSF examination, repeated for suspected brain infection, excluded viral or opportunistic infections; neither OCB or increased intrathecal Ig synthesis were detected. Non conventional 3 T MRI analysis, with DIR sequences, showed significant cortical/iuxtacortical lesion load (Fig. 1b): 23 GM lesions (GM lesion volume: 2438.0 cm^3^), of which 6 probably intracortical, were detected. After a new optical relapse with multiple active brain lesions, plasma exchange therapy allowed recovery of visual impairment.

In May 2014, after a sensitive relapse, the patient started cyclophosphamide treatment (800 mg/m^2^ i.v. monthly) which she carried on until March 2015. Thereafter for 3 months she was given oral dimethyl-fumarate, without effects in reducing disease activity (Fig. 1b). At that time the patient showed severe ataxic gait with reduced vibratory and tactile sensation, EDSS was 4.5. A specific battery of cognitive tests [7, 8] revealed cognitive impairment, in particular with regards to memory and attention domains. In June 2016 she started Alemtuzumab therapy. At the last follow-up in December 2017, no adverse effects nor disease activity were noticed; neurological examination showed moderate ataxic gait and patient had resumed most of her usual activities.

**Fig. 2:**
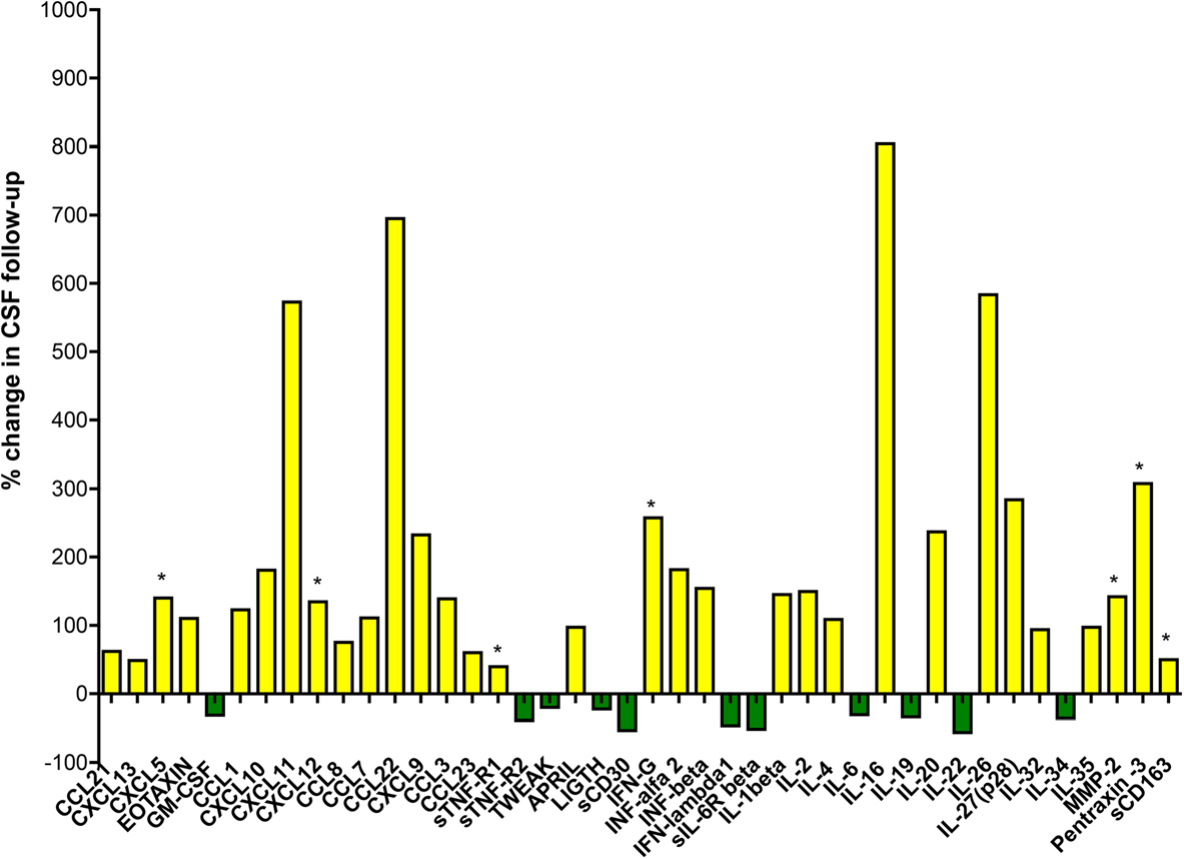
Protein analysis. Percentage of the changes in cytokines levels of the samples obtained at time of diagnosis (t0) and after 7 years of follow up (t1) CSF. Yellow bars indicate % increased levels while green bars indicate decreased levels of the inflammatory molecules. In particular, asterisks indicate CSF biomarkers of cortical damage

Advanced protein analysis technology (Bio-Plex System, BioRad) was used to assess the presence and levels of 70 inflammatory/cytotoxic proteins in paired serum and CSF samples obtained from the examined MS case at the time of diagnosis (2006, t0) and in 2013 (t1), following the procedures previously optimized [4]. By using t test and the non-parametric Mann-Whitney test to detect potential significant differences (*p*-value < 0.05) in protein levels in the two CSF samples collected at t0 and t1, we found that at time of diagnosis 42 inflammatory molecules overexpressed (at least 2 fold change, *p* < 0.05) in CSF of our patient (Fig. 2) respect to a control group of 26 patients including 12 with non-inflammatory neurological diseases and 14 with other inflammatory neurological diseases previous examined in detail [4]. Interestingly, with the exception of TNF, BAFF and IL8 that remained unchanged, 11 out of these 42 inflammatory molecules, including CXCL13, CXCL12, IFNγ, TNF, sTNFR1, IL8, sCD163, APRIL, BAFF, pentraxin III and MMP2, were found significantly increased (at least 2 fold change, *p* < 0.05) after 7 years (Fig. 2, Additional file 1: Table S1). On the other hand we found at the second time point (t1) a decrease in the levels of GM-CSF, sTNFR2, TWEAK, LIGTH, sCD30, IFNλ1, sIL6-Rβ, IL6, IL19, IL22 and IL34 (Fig. 2).

On the contrary, no substantial changes in levels of the same inflammatory proteins have been observed in the two consecutive paired serum samples and when comparing these with a pool of control sera (data not shown).

In addition, by using ELISA procedure previously optimized for the analysis of CSF protein expression of neurofilament light chains (Nf-L) [4], we found significant increase of CSF levels of Nf-L from t0 (4400 pg/ml) to t1 (6300 pg/ml).

## Discussion and conclusions

In this report, we have described the concurrence of clinical and MRI activity with the increase over time of specific CSF inflammatory markers.

Our data supports the hypothesis that an intrathecal inflammatory profile, including in particular CXCL13, IFNγ, TNF, CXCL12, LIGHT, IL6 and IL10, present at the time of diagnosis, could persist and characterize all over the disease evolution, being not easily targeted by different treatments administered. Together with increased Nf-L CSF levels over time, characterization of this profile could help the identification of prognostic inflammatory and neurodegenerative biomarkers of disease phenotypes and evolution [3, 4]. Interestingly, high disease activity was not strongly associated to significant disability accumulation in first years of the disease when also no oligoclonal bands or significant intrathecal Ig synthesis were detected, which is an immunological property of a small proportion of patients.

Among the molecular changes observed in the CSF during the follow-up period (Fig. 2), the increased levels of IL16, sCD163, sTNFR1, IFNα2, IFNβ, IFNγ in the CSF suggest that both innate and adaptive immune responses persist intrathecally and may contribute to MS pathogenesis. It is furthermore worth mentioning that while high CSF levels of TNF protein persist over time, the level of the pro-inflammatory and pro-apoptotic receptor, sTNFR1, potential marker of M1 macrophage phenotype, significantly increase, while the level of the anti-inflammatory and pro-survival receptor, sTNFR2, possible marker of M2 macrophage phenotype, decrease. Despite the limitations of a single case analysis, these data suggest that, despite the several immunomodulatory treatments, a shift in the balance between pro- and anti-inflammatory pathways may induce and/or enhance disease evolution and severity in some MS patient; we underline the usefulness of CSF assessment, combined with clinical and MRI follow-up, to identify the specific immune-inflammatory profile involved in the disease evolution of each MS patient.

## Additional file

Additional file 1: **Table S1.** Values of evaluated CSF proteins in the two consecutive samples obtained respectively in 2006 (t0) and 2013 (t1). Double arrows indicate significant changes (*p* < 0.05), measured by non-parametric Mann-Whitney test. Concentrations of the molecules are expressed as pg/ml/mg. (DOCX 33 kb)

## Data Availability

The complete data are available from the corresponding author on reasonable request.

## Abbreviations

APRIL: A proliferation-inducing ligand
BAFF: B cell activating factor
CCL 22: C-C motif chemokine 22
CCL23: C-C motif chemokine 23
CCL3: C-C motif chemokine 3
CCL7: C-C motif chemokine 7
CSF: Cerebrospinal fluid
CXCL10: C-X-C motif chemokine 10
CXCL11: C-X-C motif chemokine 11
CXCL12: C-X-C motif chemokine 12
CXCL13: C-X-C motif chemokine 13
CXCL5: C-X-C motif chemokine 5
CXCL9: C-X-C motif chemokine 9
DIR: Double Inversion Recovery
EDSS: Expanded disability status scale
ELISA: Enzyme-linked immunosorbent assay
FLAIR: Fluid Attenuated Inversion Recovery
GM: Gray matter
GM-CSF: Granulocyte-macrophage colony-stimulating factor
IFN alfa2: Interferon alfa-2
IFN beta: Interferon beta
IFNγ: Interferon gamma
IFNλ1: Interferon lambda-1
IFNλ2: Interferon lambda-2
Ig: Immunoglobulin
IL10: Interleukin-10
IL16: Interleukin-16
IL19: Interleukin-19
Il1beta: Interleukin 1beta
IL2: Interleukin-2
IL20: Interleukin-20
IL22: Interleukin-22
IL26: Interleukin-26
IL27: Interleukin-27
IL32: Interleukin 32
IL34: Interleukin-34
IL35: Interleukin-35
IL4: Interleukin-4
IL6: Interleukin-6
IL8: Interleukin-8
LIGTH: Tumour necrosis factor superfamily member 14
LP: lumbar puncture
MMP2: Matrix metallopeptidase 2
MS: Multiple sclerosis
Nf-L: Neurofilament light chains
OCB: Oligoclonal bands
sCD163: Soluble-CD163 (Cluster of Differentiation 163)
sCD30: Soluble-CD30 (Cluster of Differentiation 30)
sIL6-Rβ: Soluble-Interleukin-6 receptor beta
sTNFR1: Soluble- tumour necrosis factor-receptor 1
sTNFR2: Soluble- tumour necrosis factor-receptor 2
sTNFR2: Soluble- tumour necrosis factor-receptor 2
TNF: Tumour necrosis factor alpha
TWEAK: TNF-related weak inducer of apoptosis
