# Supplementary material for: Increase of CSF inflammatory profile in a case of highly active multiple sclerosis
Source: BMC Neurol. 2019 Sep 26;19:231. doi: 10.1186/s12883-019-1455-7 (PMC6764139; doi:10.1186/s12883-019-1455-7)
Supplement: Supplementary file 1 — Table S1. Values of evaluated CSF proteins in the two consecutive samples obtained respectively in 2006 (t0) and 2013 (t1). Double arrows indicate significant changes (p < 0.05), measured by non-parametric Mann-Whitney test. Concentrations of the molecules are expressed as pg/ml/mg. (DOCX 33 kb) [file 12883_2019_1455_MOESM1_ESM.docx]

**Table S1** Values of evaluated CSF proteins in the two consecutive samples obtained respectively in 2006 (t0) and 2013 (t1). Double arrows indicate significant changes (p< 0.05), measured by non-parametric Mann-Whitney test. Concentrations of the molecules are expressed as pg/ml/mg.

| **Molecules** | **t0** | **t1** | **Change** | **Type** | **Site of production** |
| --- | --- | --- | --- | --- | --- |
| APRIL/TNFSF13 | 25511,61 | 50873,64 | ↑ | Pro-inflammatory | Monocytes/T-cells |
| BAFF/TNFSF13B | 6717,59 | 5433,47 | - | Pro-inflammatory | B-Cells |
| BCA-1/CXCL13 | 21,60 | 32,61 | ↑ | Pro-inflammatory | Monocyte /dentridic cells |
| sCD30/TNFRSF8 | 500,35 | 277,30 | ↓ | Pro-inflammatory | T-cells |
| sCD163 | 43506,91 | 66170,65 | ↑↑ | Pro-inflammatory | macrophages |
| Chitinase 3-like 1 | 192611,90 | 22002,62 | ↑ | Pro-Inflammatory | macrophages |
| 6Ckine/CCL21 | 450,07 | 739,78 | ↑ |  | dendritic cells/T-cells |
| CTACK/CCL27 | - | - |  | Pro-Inflammatory | T-cells |
| ENA-78/CXCL5 | 48,74 | 118,16 | ↑↑ | Pro-Inflammatory | Astocytes/ microglia |
| Eotaxin/CCL11 | 7,80 | 16,58 | ↑ | Pro-Inflammatory | eosinophiles |
| Eotaxin-2/CCL24 | 35,13 | 38,69 | ↑ | Pro-Inflammatory | eosinophiles |
| Eotaxin-3/CCL26 | 9,45 | 12,96 | ↑ | Pro-Inflammatory | eosinophiles |
| Fractalkine/CX3CL1 | 143,25 | 161,04 | ↑ | Pro-Inflammatory | Microglia/neurons |
| GCP-2/CXCL6 | - | - |  | Pro-Inflammatory | oligodendrocytes |
| GM-CSF | 57,02 | 19,31 | ↓ | Pro-Inflammatory | B-Cell / T-Cell |
| Gro-a/CXCL1 | 46,95 | 47,92 | ↑ | Pro-Inflammatory | oligodendrocytes |
| Gro-b/CXCL2 | 0,00 | 12,87 | ↑ | Pro-Inflammatory | oligodendrocytes |
| I-309/CCL1 | 22,90 | 51,53 | ↑ | Pro-Inflammatory | monocytes/ macrophages/T-cell |
| IFN-a2 | 5,20 | 14,76 | ↑ | Anti-inflammatory | T-cell |
| IFN-b | 20,04 | 51,41 | ↑ | Anti-Inflammatory | T-cell |
| IFN-g | 2,60 | 9,36 | ↑ | Pro-Inflammatory | T-cell(Th1) |
| IL-1b | 0,25 | 0,37 | ↑ | Pro-Inflammatory | dentridic cells/mast cells/B-cells |
| IL-2 | 0,77 | 1,94 | ↑ | Pro-Inflammatory | T-cells |
| IL-4 | 8,41 | 17,74 | ↑ | Anti-inflammatory | T-cells/dendritic cells |
| IL-6 | 13,38 | 4,41 | ↓ | Anti-inflammatory | T-cells/dendritic cells/microglia |
| sIL-6Ra | 3219,14 | 4187,96 | ↑ | Anti-inflammatory | T-cells/ macrophages, neutrophils |
| sIL-6R b | 40265,88 | 21599,36 | ↓ | Anti-inflammatory | Neurons/astrocytes/ microglia/ endothelial cell |
| IL-8 | 23,40 | 20,15 | - | Pro-Inflammatory | Monocytes/macrophages |
| IL-10 | 4,18 | 4,36 | - | Anti-inflammatory | T-cells/astocytes |
| IL-11 | 0,00 | 0,49 | ↑ | Anti-inflammatory | Astrocytes |
| IL-12(p40) | 0,00 | 0,10 | ↑ | Pro-Inflammatory | APC |
| IL-12(p70) | 0,78 | 0,13 | ↓ | Pro-Inflammatory | B-cells/ dendritic cells/ macrophages |
| IL-16 | 16,19 | 146,65 | ↑↑ | Pro-Inflammatory | T-cells (Th1) |
| IL-19 | 68,84 | 24,84 | ↓ | Anti-Inflammatory | Monocytes |
| IL-20 | 3,12 | 10,58 | ↑ | Pro-Inflammatory | Monocytes |
| IL-22 | 24,76 | 14,53 | ↓ | Anti-Inflammatory | T-cells(Th17-22) |
| IL-26 | 67,82 | 465,16 | ↑↑ | Pro-Inflammatory | T-cells(Th17) |
| IL-27(p28) | 129,37 | 499,96 | ↑↑ | Pro-Inflammatory | Astrocytes/ microglia/macrophages |
| IL-28A/IFN-lamba2 | 116,90 | 126,11 | ↑ | Anti-Inflammatory | Dendritic cells |
| IL-29/IFN-lamba1 | 12,02 | 5,85 | ↓ | Anti-Inflammatory | Dendritic cells |
| IL-32 | 57,47 | 112,62 | ↑ | Pro-Inflammatory | T-cells |
| IL-34 | 202,80 | 76,87 | ↓ | Anti-Inflammatory | Macrophages/neurons |
| IL-35 | 150,49 | 300,17 | ↑ | Pro-Inflammatory | B-cells |
| IP-10/CXCL10 | 173,84 | 492,49 | ↑ | Pro-Inflammatory | T-cells |
| I-TAC/CXCL11 | 0,42 | 2,83 | ↑ | Anti-Inflammatory | T-cells(T-reg) |
| LIGHT/TNFSF14 | 1342,94 | 331,36 | ↓↓ | Anti-Inflammatory | T-cells/ Dendritic cells |
| MCP-1/CCL2 | 360,13 | 317,69 | ↓ | Pro-Inflammatory | Astrocytes/Monocytes |
| MCP-2/CCL8 | 5,69 | 10,09 | ↑ | Pro-Inflammatory | Astrocytes/Monocytes |
| MCP-3/CCL7 | 9,76 | 20,83 | ↑ | Pro-Inflammatory | Astrocytes/Monocytes |
| MCP-4/CCL13 | 1,55 | 1,90 | ↑ | Pro-Inflammatory | Monocytes/ macrophages (M2)/ dendritic cells/ T-cells |
| MDC/CCL22 | 5,10 | 40,68 | ↑↑ | Pro-Inflammatory | Macrophages (M2) |
| MIF | 3487,55 | 559,05 | ↓↓ | Pro-Inflammatory | oligodendrocytes |
| MIG/CXCL9 | 18,74 | 62,75 | ↑ | Pro-Inflammatory | Astrocytes |
| MIP-1a/CCL3 | 3,17 | 7,65 | ↑ | Pro-Inflammatory | Macrophage/ T-cells/ dendritic cells |
| MIP-1d/CCL15 | 222,86 | 219,74 | ↓↓ | Pro-Inflammatory | Dendritic cells/ T-cells |
| MIP-3a/CCL20 | 0,00 | 1,01 | ↑ | Pro-Inflammatory | T-cells/ monocytes |
| MIP-3b/CCL19 | 67,23 | 94,28 | ↑ | Pro-Inflammatory | B-cells |
| MPIF-1/CCL23 | 3,61 | 5,86 | ↑ | Pro-Inflammatory | T-cells(Th1) |
| MMP-1 | 509,20 | 413,36 | - | Pro-Inflammatory | Monocytes/Macrophages/Endotelial cells |
| MMP-2 | 335,18 | 818,34 | ↑ | Pro-Inflammatory | Monocytes/Macrophages/Endotelial cells |
| MMP-3 | - | - | - | Pro-Inflammatory | Monocytes/Macrophages/Endotelial cells |
| Osteocalcin | 488,24 | 413,07 | - |  |  |
| Osteopontin | 57720,14 | 67934,87 | - | Pro-Inflammatory | Micorglia/neurones/ T cells/ dendritic cells/ macrophages/ natural killer |
| Pentraxin-3 | 107,88 | 442,35 | ↑ | Pro-Inflammatory | Dendritic cells/macrophages |
| SCYB16/CXCL16 | 1124,16 | 814,76 | ↓ | Anti-inflammatory | Leucocytes/endotelial cells |
| SDF1a+b/CXCL12 | 1303,70 | 1788,65 | ↑ | Pro-Inflammatory | Astrocytes/T-cells |
| TARC/CCL17 | - | - |  | Pro-Inflammatory | B-cells/T-cells/macrophages |
| TECK/CCL25 | 5,74 | 107,16 | ↑ |  |  |
| TNF-a | 18,83 | 21,13 | - | Pro-Inflammatory | Microglia/T-cells(Th22)/macrophages(M1)/B-cells |
| sTNF-R1 | 6082,47 | 8627,19 | ↑↑ | Pro-Inflammatory | Microglia/T-cells(Th22)/macrophages(M1)/Bcells |
| sTNF-R2 | 1647,78 | 675,66 | ↓↓ | Anti-Inflammatory | Microglia/T-cells(Th22)/macrophages(M1)/Bcells |
| TWEAK | 2469,02 | 547,93 | ↓↓ | Pro-Inflammatory | monocytes/macrophage/microglia |
| TSLP | 25,84 | 28,23 | ↑ | Pro-Inflammatory | T-cells(Th17) |
